# Editorial: Vaccines and molecular therapeutics for tuberculosis

**DOI:** 10.3389/fimmu.2026.1809428

**Published:** 2026-03-10

**Authors:** Suraj B. Sable, Vijayakumar Velu, Andreas Kupz

**Affiliations:** 1 Indepent Researcher, Atlanta, GA, United States; 2 Division of Pathology and Laboratory Medicine, Emory University School of Medicine and Division of Microbiology and Immunology, Emory National Primate Research Center, Emory Vaccine Center,, Atlanta, GA, United States; 3 Australian Institute of Tropical Health and Medicine, James Cook University, Cairns, QLD, Australia

**Keywords:** “site-aware” tuberculosis vaccination, BCG - Bacille Calmette-Guérin vaccine, correlates of protection, granuloma topography, host-directed therapies, metabolism-linked antigenicity and immunogenicity, *Mycobacterium tuberculosis*, vaccine platform and delivery technologies

## Introduction

**Figure 1:**
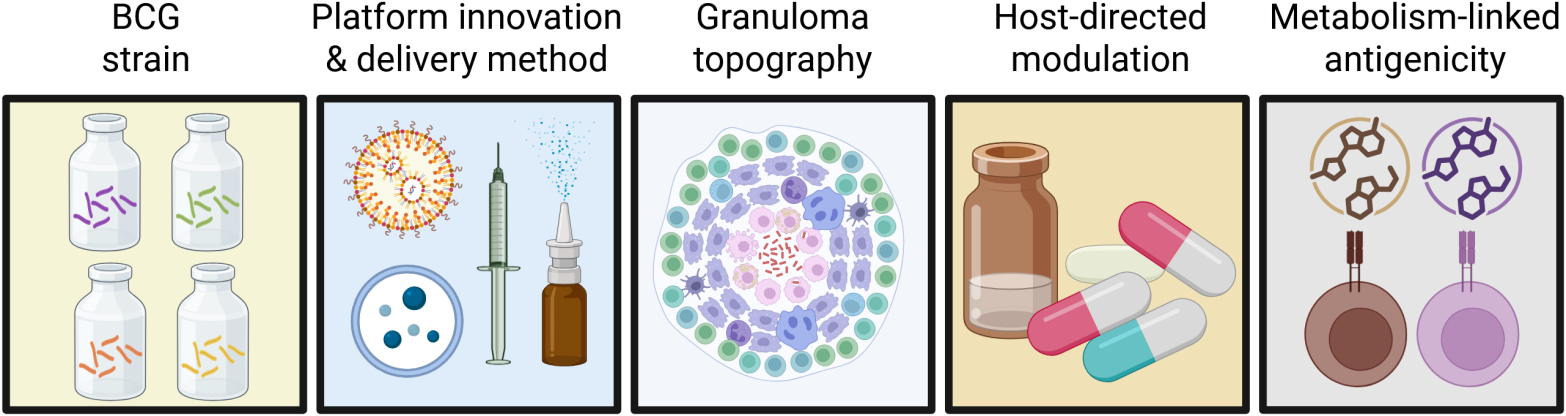
Factors influencing tuberculosis vaccine and therapy success. The articles published in this Research Topic provide evidence that progress in the development of novel vaccines and molecular therapeutics for tuberculosis likely depend on several factors. These include understanding and dissecting differences among licensed BCG strains; developing further innovations in platform technologies and delivery methods; delineating the diversity of granuloma microenvironments; inclusion of targeted host-directed modulation; and further advances in harnessing metabolism-linked antigenicity. Created in BioRender. Kupz, A. (2026) https://BioRender.com/1yt8jw3.

Tuberculosis (TB) vaccinology is at a critical juncture. While Bacille Calmette–Guérin (BCG) has been used as a vaccine for a century and offers strong protection against severe disseminated disease in infants, its effectiveness against adult pulmonary TB varies significantly (1, 2). Currently, a growing pipeline of vaccines, including protein-adjuvant boosters, acellular mycobacterial products, nanoparticle- and nucleic acid platforms, has demonstrated robust immune responses, though the precise correlates of protection and indicators of immunity remain unclear (3). The collection of articles published in the Research Topic “Vaccines and Molecular Therapeutics for Tuberculosis” indicates that the field isn’t lacking new vaccine ideas; rather, it is still exploring how to induce protective immunity within lung microenvironments where *Mycobacterium tuberculosis* (*Mtb*) persists. These studies emphasize that vaccine success likely depends on factors beyond antigen selection, such as differences among BCG strains, heterologous immune effects, lung immunoregulation, and the diversity of granuloma microenvironments (**Figure 1**). A common theme emerged: future TB vaccines will likely involve combination strategies that activate effective immunity at the infection site while addressing immunosuppression and lesion heterogeneity. We advocate designing “site-aware” vaccines that generate protective responses directly at the exposure site, along with targeted host-directed therapies employing molecular therapeutics for TB.

## BCG vaccine

Torracinta et al. conducted a systematic review of the current literature on the immune mechanisms mediating the heterologous effects of BCG vaccination. System-level analyses of BCG’s heterologous effects reveal that BCG is not a single, standardized entity. Its strain background and manufacturing history influence innate and adaptive immune responses, which are also affected by host factors such as age at vaccination, sex, baseline inflammatory status, and prior microbial exposure. This perspective further supports previous pre-clinical research (4) and changes how we should view BCG, not just as a comparator but also as a variable biological starting point. In trial design and preclinical studies, it is essential to specify the BCG strain, document the context, and interpret boosting outcomes, acknowledging that different BCG baselines may respond differently to boosting. For mechanistic research, it emphasizes the importance of measuring trained immunity and innate immune programming alongside traditional T cell responses (5).

## Platform innovation is necessary but insufficient

Several strategies involving BCG demonstrate that achieving measurable benefits depends on enhancing immune quality rather than simply increasing the magnitude of the immune response (6). Post-BCG immune enhancement through immunotherapy with B cell-activating factor (BAFF) or a proliferation-inducing ligand (APRIL), as reported by Xie et al., is noteworthy because it directly targets the immune system, boosting B cell and T cell functions, reducing the pro-inflammatory response, and improving protection during experimental challenges. Similarly, in a study by Ouyang et al., a heterologous boost with a fusion protein of Ag85B, Rv2660, and MPT-70 (ARM) in a CPG-ODN adjuvant suggests that better immunity may also require effective immunoregulation in the lung post-challenge: protection is associated with qualitative improvements in effector potential, not just higher levels of a single cytokine.

Yamaguchi et al. underscore the importance of BCG-derived membrane vesicles as novel acellular vaccine candidates. Delivering nutrient starvation-enriched vesicles intranasally enhances mucosal and trained innate responses and improves anti-mycobacterial immunity. These vesicle-based strategies are appealing because they retain complex mycobacterial lipids and proteins without the risks associated with live, replicating organisms, and they naturally target the airways, the primary site of infection, as a key mechanism of infection and immune modulation.

Nucleic-acid vaccines offer a key advantage: rapid development and flexibility (7, 8). A thorough review of DNA and RNA TB vaccines by Kazakova et al. highlights their promise but also identifies challenges, such as differences in antigen selection, delivery methods, animal models, and measurement standards, that make cross-study comparisons difficult. In this context, lipid-nanoparticle (LNP) mRNA vaccines that encode multiple *Mtb* antigens act as a proof of concept. A mRNA vaccine study by De Voss et al. shows that both single-antigen and five-antigen mixed formulations generate strong cellular and humoral immune responses and reduce bacterial load in a mouse *Mtb* challenge model. However, when used as boosters after BCG vaccination, these mRNA vaccines sustain immune responses but do not significantly improve protection beyond BCG alone. Importantly, combining multivalent mRNA vaccines with a mucosal chimpanzee adenoviral vector in a heterologous prime-boost approach enhances protection, suggesting that platform synergy and delivery method are as important as antigen choice.

## Granuloma topography is a design constraint

A review by Krueger et al. underscores the importance of understanding granuloma biology to address a key knowledge gap. Spatial and microenvironmental research now describes the tuberculous granuloma as a complex mosaic of distinct cellular neighborhoods, nutrient and oxygen gradients, and immune ecosystems unique to each lesion that evolve over time (9–12). These features influence how antigens are presented, where lymphocytes are located, macrophage antimicrobial activity, and drug penetration into tissues. Although vaccine development presents significant challenges, success is possible. Relying solely on blood measurements and average lung data may miss the critical question: Can vaccine-induced effectors access granulomas, remain within them, and work effectively in the niches where *Mtb* persists?

If microenvironments are regarded as the battlefield, vaccine evaluation should focus more on that context. Tissue-relevant endpoints should include spatial measurements of effector cell infiltration into infected macrophage areas, phenotypes of antigen-presenting cells, and the balance between inflammatory and suppressive myeloid activities within lesions. Granuloma research further underscores the need for adjunct host-directed strategies. Improving the lesion environment could increase the likelihood that robust systemic immunity translates into effective local immunity.

## Host−directed modulation can unlock vaccine efficacy

An example of a modifiable barrier in the Research Topic is vaccine-induced myeloid-derived suppressor cells (MDSCs) as reported by Aintablian et al. Heat-killed *Mtb* immunization in mice led to a significant increase in monocytic MDSCs with suppressive function. Despite this, animals still demonstrated protection against a subsequent mycobacterial (BCG) challenge, which involved dendritic cell activation and the expansion of mycobacteria-specific T cells. Notably, removing CCR2^+^ monocytic cells genetically or treating with all-trans retinoic acid (ATRA) decreased MDSC levels and further lowered bacterial burden, while enhancing dendritic cell and T cell activation.

The main point is not to suggest that every TB vaccine must be combined with ATRA. Instead, it emphasizes that vaccines can activate suppressive myeloid programs that coexist with protective immunity and can be therapeutically modified to improve control. In TB vaccinology, this introduces a combination approach like oncology strategies: vaccine platforms that elicit antigen-specific responses may require adjuncts that reduce local suppression or normalize lesion architecture, enabling adaptive effectors to more effectively target infected cells.

## Metabolism−linked antigenicity widens the immunological canvas

The final frontier exists at the intersection of metabolism and immunity (13, 14). A review by Oketade et al. on flavin and deazaflavin biosynthesis emphasizes that conserved metabolic pathways are vital for mycobacterial health and can be detected immunologically via MR1-presented ligands. MR1-restricted T cells, including mucosal-associated invariant T (MAIT) cells, are located at airway surfaces where early *Mtb* exposure occurs, and their activation depends on metabolic intermediates rather than peptides (15). This suggests the potential to develop vaccine strategies that extend beyond traditional peptide antigens and to explore whether metabolic antigens can trigger protective, tissue-resident immune responses.

An important caveat is that some antimycobacterial prodrugs depend on deazaflavin biology for activation (16–18), and because metabolic intermediates can act as MR1 agonists or antagonists, drug-vaccine interactions (including during adjunct host-directed therapies) might alter antigenicity itself. Incorporating immunometabolism into TB vaccine strategies is not just about adding complexity; it’s an opportunity to develop interventions that are mechanistically aligned with both immune responses and microbial physiology.

## A practical agenda for the next phase

A productive way forward for the development of improved TB vaccines involves aligning platforms, locations, and adjunctive therapies (**Figure 1**). Platforms include not only advanced recombinant BCG strains and other live-attenuated vaccines, but also rational *Mtb* immunogens, improved protein-adjuvant formulations, nanoparticles, and flexible mRNA constructs that can be quickly adapted as antigen priorities evolve. Location considerations involve high-dimensional evaluation of lung and granuloma microenvironments through spatial multi-omics, imaging, and *ex vivo* granuloma-like models to identify lesion-specific markers and detect early signs of microenvironment failure or vaccine efficacy. Adjunct therapies should include host-targeted immune modulators that reduce suppressive myeloid programs or normalize lesion structure, thereby providing strategic support for vaccination efforts rather than serving as secondary options (19–21).

The main message is straightforward: TB vaccines will be evaluated not only by the immunity they produce but also by their ability to provoke immune responses in diverse, metabolically limited, and often suppressive environments in which *Mtb* persists. Using site-specific design and strategic combinations of vaccination with host-directed modulation provides a practical way to turn current immunogenicity successes into lasting protection.
